# Understanding the contribution of UK public health research to clinical guidelines: a bibliometric analysis

**DOI:** 10.12688/f1000research.18757.1

**Published:** 2019-07-15

**Authors:** Susan Guthrie, Gavin Cochrane, Advait Deshpande, Benoit Macaluso, Vincent Larivière

**Affiliations:** 1 RAND Europe, Westbrook Centre, Milton Road, Cambridge , CB4 1YG , UK; 2 Observatoire des sciences et des technologies, University of Quebec at Montreal, 1205 St. Denis Street, Montréal, Québec, H2X 3R9, Canada; 3 École de bibliothéconomie et des sciences de l’information, University of Montreal, 3150 rue Jean-Brillant, Montréal, Québec, H3T 1N8, Canada

**Keywords:** Public health, bibliometrics, guidelines, research funding, UK

## Abstract

**Background:** There is an increasing need to understand the wider impacts of research on society and the economy. For health research, a key focus is understanding the impact of research on practice and ultimately on patient outcomes. This can be challenging to measure, but one useful proxy for changes in practice is impact on guidelines.

**Methods:** The aim of this study is to map the contribution of UK research and UK research funders to the National Institute for Health and Clinical Excellence (NICE) public health guidelines, understanding areas of strengths and weakness and the level of collaboration and coordination across countries and between funders. The work consisted of two main elements: analysis of the references cited on NICE guidelines and interviews with experts in public health.

**Results:** Across the papers cited on 62 NICE public health guidelines, we find that 28% of the papers matched include at least one UK affiliation, which is relatively high when compared to other health fields. In total, 165 unique funders were identified with more than three acknowledgements, based in 20 countries. 68% of papers which acknowledge funding cite at least one UK funder, and NIHR is the most highly cited funder in the sample.

**Conclusions:** The UK makes an important contribution to public health research cited on NICE PH guidelines, although the research does not appear to be bibliometrically distinct from other research sectors, other than having a relatively low level of international collaboration. However, the extent to which NICE public health guidelines reflect practice at the local authority level is less clear. More research is needed to understand the sources of evidence to support public health decision making at the local level and how NICE guidance can be made more applicable, timely and accessible in this new context.

## Introduction

There is an increasing need to understand the wider impacts of research on society and the economy. In the UK, this is reflected in the introduction of impact case studies into the national assessment system, the Research Excellence Framework, through which core funding is allocated to universities. More widely, there are moves to understand and demonstrate the impact of research outside the academic sphere. This reflects the wider political climate. Economic austerity and increased demands on public expenditure to provide direct services put pressure on research budgets and there is an increased need to show why investment in research is worthwhile.

For health research, a key focus is understanding the impact of research on practice and ultimately on patient outcomes. This can be challenging to measure, but one useful proxy for changes in practice is impact on guidelines. In the UK in particular, evidence that influences NICE guidelines can make a strong case for an impact on patient treatment in the NHS. This is an approach that has been used previously to analyse the way research affects practice in a number of fields ^1– 3^. However, the picture for public health is more nuanced, partly since interventions may often not be delivered through the NHS. In this study, we explore the impact of UK public health research on NICE public health guidelines.

The aims of this article are: to understand the importance of UK research and UK research funders in developing the evidence for the guidance provided, relative to other fields and across areas of public health; to compare the body of evidence that underpins NICE guidelines in this field to other areas; to consider the appropriateness of this approach in the public health context; and to consider the extent and way in which guidelines shape practice in this field.

## Methods

The aim of this study is to map the contribution of UK research and UK research funders to NICE public health guidelines, understanding areas of strength of weakness and the level of collaboration and coordination across countries and between funders. The work consisted of two main elements: analysis of the references cited on NICE guidelines and interviews with experts in public health. We discuss each of these methods in more detail in the remainder of this section.

### Analysis of references cited on NICE guidelines

We identified 62 currently active NICE public health guidelines from the NICE website (data extracted on 26/09/2016 ). The guidelines included are listed in Table 1. These guidelines were grouped into ten field categories, as indicated in Table 1, by two members of the team (GC, SG) based primarily on their titles, though a brief review of content was also conducted where necessary (e.g. if the title was ambiguous).

**Table 1.** List of guidelines included in the study.

No.	Title	Health area	Year
1	Smoking: brief interventions and referrals	Addiction, substance abuse	2006
2	Sexually transmitted infections and under-18 conceptions: prevention	Sexual Health	2007
3	Substance misuse interventions for vulnerable under 25s	Addiction, substance abuse	2007
4	Smoking: workplace interventions	Addiction, substance abuse	2007
5	Behaviour change: general approaches	General	2007
6	Alcohol: school-based interventions	Addiction, substance abuse	2007
7	Physical activity and the environment	Physical activity	2008
8	Stop smoking services	Addiction, substance abuse	2008
9	Maternal and child nutrition	Obesity & Nutrition	2008
10	Social and emotional wellbeing in primary education	Mental Health and Wellbeing	2008
11	Physical activity in the workplace	Physical activity	2008
12	Smoking: preventing uptake in children and young people	Addiction, substance abuse	2008
13	Cardiovascular disease: identifying and supporting people most at risk of dying early	Cardiovascular	2008
14	Mental wellbeing in over 65s: occupational therapy and physical activity interventions	Mental Health and Wellbeing	2008
15	Physical activity for children and young people	Physical activity	2009
16	Workplace health: long-term sickness absence and incapacity to work	General	2009
17	Social and emotional wellbeing in secondary education	Mental Health and Wellbeing	2009
18	Immunisations: reducing differences in uptake in under 19s	General	2009
19	Mental wellbeing at work	Mental Health and Wellbeing	2009
20	Smoking prevention in schools	Addiction, substance abuse	2010
21	Alcohol-use disorders: prevention	Addiction, substance abuse	2010
22	Cardiovascular disease prevention	Cardiovascular	2010
23	Smoking: stopping in pregnancy and after childbirth	Addiction, substance abuse	2010
24	Weight management before, during and after pregnancy	Obesity & Nutrition	2010
25	Looked-after children and young people	General	2010
26	Unintentional injuries: prevention strategies for under 15s	General	2010
27	Unintentional injuries in the home: interventions for under 15s	General	2010
28	Unintentional injuries on the road: interventions for under 15s	General	2010
29	Skin cancer prevention	Cancer	2011
30	HIV testing: increasing uptake in black Africans	Sexual Health	2011
31	HIV testing: increasing uptake in men who have sex with men	Sexual Health	2011
32	Type 2 diabetes prevention: population and community-level interventions	Diabetes	2011
33	Healthcare-associated infections: prevention and control	General	2011
34	Type 2 diabetes: prevention in people at high risk	Diabetes	2012
35	Smokeless tobacco: South Asian communities	Addiction, substance abuse	2012
36	Social and emotional wellbeing: early years	Mental Health and Wellbeing	2012
37	Physical activity: walking and cycling	Physical activity	2012
38	Obesity: working with local communities	Obesity & Nutrition	2012
39	Hepatitis B and C testing: people at risk of infection	Sexual Health	2012
40	Physical activity: brief advice for adults in primary care	Physical activity	2013
41	Smoking: harm reduction	Addiction, substance abuse	2013
42	BMI: preventing ill health and premature death in black, Asian and other minority ethnic groups	General	2013
43	Weight management: lifestyle services for overweight or obese children and young people	Obesity & Nutrition	2013
44	Smoking: acute, maternity and mental health services	Addiction, substance abuse	2013
45	Behaviour change: individual approaches	General	2014
46	Domestic violence and abuse: multi-agency working	General	2014
47	Contraceptive services for under 25s	Sexual Health	2014
48	Needle and syringe programmes	Addiction, substance abuse	2014
49	Weight management: lifestyle services for overweight or obese adults	Obesity & Nutrition	2014
50	Physical activity: exercise referral schemes	Physical activity	2014
51	Oral health: local authorities and partners	Oral Health	2014
52	Vitamin D: increasing supplement use in at-risk groups	General	2014
53	Excess winter deaths and illness and the health risks associated with cold homes	General	2015
54	Preventing excess weight gain	Obesity & Nutrition	2015
55	Workplace health management practices	General	2015
56	Dementia, disability and frailty in later life – mid-life approaches to delay or prevent onset	Mental Health and Wellbeing	2015
57	Oral health promotion: general dental practice	Oral Health	2015
58	Older people: independence and mental wellbeing	Mental Health and Wellbeing	2015
59	Sunlight exposure: risks and benefits	General	2016
60	Community engagement: improving health and wellbeing and reducing health inequalities	General	2016
61	Oral health for adults in care homes	Oral Health	2016
62	Harmful sexual behaviour among children and young people	Sexual Health	2016

The references were extracted from the guidelines manually into a database, producing a total of 31,409 references across all guidelines. It should be noted that many of these guidelines do not include all or, in some cases, any of the references for the evidence that underpinned their development directly. Often, separate evidence summaries are provided (also on the NICE website) for the guidelines – sometimes several for one guideline – which provide references for the underpinning evidence. We extracted the references from these evidence summaries as well as directly from the guidelines themselves.

The references were matched in the Web of Science (WoS) database. Once duplicates were removed and documents which were not journal articles excluded, we were able to produce a set of 12,706 papers from the guidelines matched in Web of Science. This is a low proportion of the total references extracted, but reflects a high level of policy documents and other non-journal material referenced in the guidelines.

For each of these papers matched in WoS, we were able to analyse the institutional affiliations of the contributing authors, based on the address information provided. We used this to look at the countries in which the research was conducted. We used a full counting approach, to allow for comparisons with previous studies in the area.

We also have the date of publication of each of the articles matched in WoS. By comparing this to the publication date of the relevant guideline, we are able to produce an estimate of the ‘knowledge cycle time’ – that is, the time between the publication of a paper and its citation on the guideline.

Finally, for a number of the publications, we were also able to extract the names of the funders supporting the research from the funding body acknowledgements (FBAs) on the publications. This is only available in WoS for publications from 2008, and even since 2008 the data is not complete, partly because authors do not always cite their funding sources completely and accurately (and sometimes not at all). These data were available for 15% of the papers matched in WoS.

Using these data sets we were able to analyse the contributions and levels of collaboration across funding bodies, countries and between institutions on the publications cited on public health guidelines, by area of public health and as a whole. Analysis was carried out using Microsoft Excel and R.

### Interviews

Interviews were conducted with eleven stakeholders engaged in public health-related decision-making and public health research in the UK. Interviewees were selected to ensure a mix of geographical settings and experiences. At the time of the interview, seven of the interview participants were directors of public health at the local authority level, tasked with responsibility for delivering public health interventions in the UK since 2013 ^4^. Four interview participants were either senior academics or researchers with significant experience of public health in the UK. In accordance with the terms of interview participation, any quotes or attributions have been anonymised where applicable.

Interviews were typically 30–60 minutes long and were conducted by telephone in a semi-structured format, based on the interview protocol provided in Table 2. An anonymised list of interviewees, along with their designation and expertise, is included in Table 3. Interviews were recorded, subject to the consent of the individuals, and summary notes written up after each interview (not full transcripts). Interviews were conducted under the principle of informed consent with interviewees informed that the recordings and notes would only be used by the study team for the purposes of the study. These files are stored on secure servers and recordings will be destroyed one year after study completion. The interview notes were analysed, and key themes and common messages extracted to generate some of the insights and the underlying context described, taking a thematic analysis approach. The emergent themes from the interviews are covered in more detail in the Discussion section of this paper.

**Table 2.** Interview protocol.

**Background** We are conducting research looking at the contribution of UK public health research to NICE public health guidelines. However, we are aware that these guidelines may not always reflect policy and commissioning at a local level, because of different needs and priorities in different contexts. Therefore, we are conducting a series of interviews with relevant informants with expertise in public health at the local level to understand how far these guidelines reflect policy and practice, and whether there are other important sources of evidence that are used or other documentation that reflects practice that we could analyse as part of our research. Do you have any questions about the project? We would like to record the interview, solely for our own internal use within the project team. We will not quote you or identify you directly without your permission. Are you happy for us to record the interview?
**Introductory questions** • I know you have many roles which might be relevant to us here, from your research to your work with NICE – could you start by telling us a little about your background and interest in this area? • What do you know about how PH priorities are developed at the local authority level, so far as those process are common? • How do those priorities then feed into commissioning decisions? • We understand that Directors of PH have overall responsibility for this process, but who else is involved from LAs, CCGs etc? How does this interaction and the process overall work – or does this vary?
**Sources of evidence for PH policy** • What are the main sources of evidence that Directors of PH and others draw on when setting PH policies or making commissioning decisions? • What role do NICE PH guidelines play? Are there particular guidelines that are more widely used or more relevant than others? • What other sources of evidence are used? Are there particular local level sources as well as wider materials? How far are decisions drawing on research evidence compared to other sources?
**Documenting PH policies and processes** • We are aware that each area now has to create joint health and wellbeing strategies. Are these the main form in which PH policies are documented, or are there other sources we should be looking at? Are the documents of interest publicly available?
**Relationship(s) between local level authorities and with national level bodies** • How do Directors of PH coordinate their work with other local level bodies, particularly CCGs (assuming they do)? Is this documented at all? • Do different LAs work together (outside of the cross LA Director of PH appointments that we are aware of), either to share good practice and learning, or potentially to commission services jointly? If so, how does this work and is it documented? • What is your view on how local level PH ties into national level efforts? ○ How effectively do you think local level PH efforts feed into national level policy-making and vice versa? ○ Is this documented? ○ Are there specific aspects of this process that you think can be improved?

**Table 3.** Interview participants.

Identifier	Description of the interview participant and the nature of their involvement with public health
INT01	Director of Public Health in the South East of England
INT02	Senior Academic expert on public health
INT03	Director of Public Health in the Midlands
INT04	Director of Public Health in the West Midlands
INT05	Director of Public Health in the East of England
INT06	Director of Public Health in the South West of England
INT07	Senior Academic Expert on public health
INT08	Senior Academic Expert on public health
INT09	Senior Academic Expert on public health
INT10	Director of Public Health in the North of England
INT11	Director of Public Health in the East of England

## Ethics and consent

The need for ethical approval was waived for this study by the RAND Human Subjects Protection Committee since interviews were conducted by individuals in their professional capacity only. Oral consent was sought from interviewees to record the interviews, with the interview recording and accompanying notes/transcripts solely for the use of the study team. Oral, rather than written, consent was sought as interviews were conducted by telephone. All participants are anonymised in the discussion in the article and we have ensured all quotes used could not identify the contributor.

## Results

In this section, we discuss the findings from the bibliometric analysis of the citation data included in the NICE guidelines. We discuss this in two main sections: (1) contribution of the UK as a provider of research, through an analysis of the institutional affiliations associated with cited papers; (2) contribution of the UK as a funder of research through an analysis of the funding body acknowledgements associated with cited papers.

### Contribution of the UK as a provider of research

The first level of analysis was to assess the distribution and co-occurrence of institutional affiliations from publications cited in the guidelines. This allows us to look at the contribution of the UK and other countries to the research conducted that underpins this set of guidelines.

We found that 28% (2,627/9,391) of the papers matched include at least one UK affiliation. Of the 2,627 papers with UK institutions, 2,114 papers (80%) contained only UK based affiliations, suggesting the proportion of international collaboration to be around 20% with non-UK based institutions. This is below the general average for international collaboration in research across the UK ^5, 6^.

**Figure 1.**
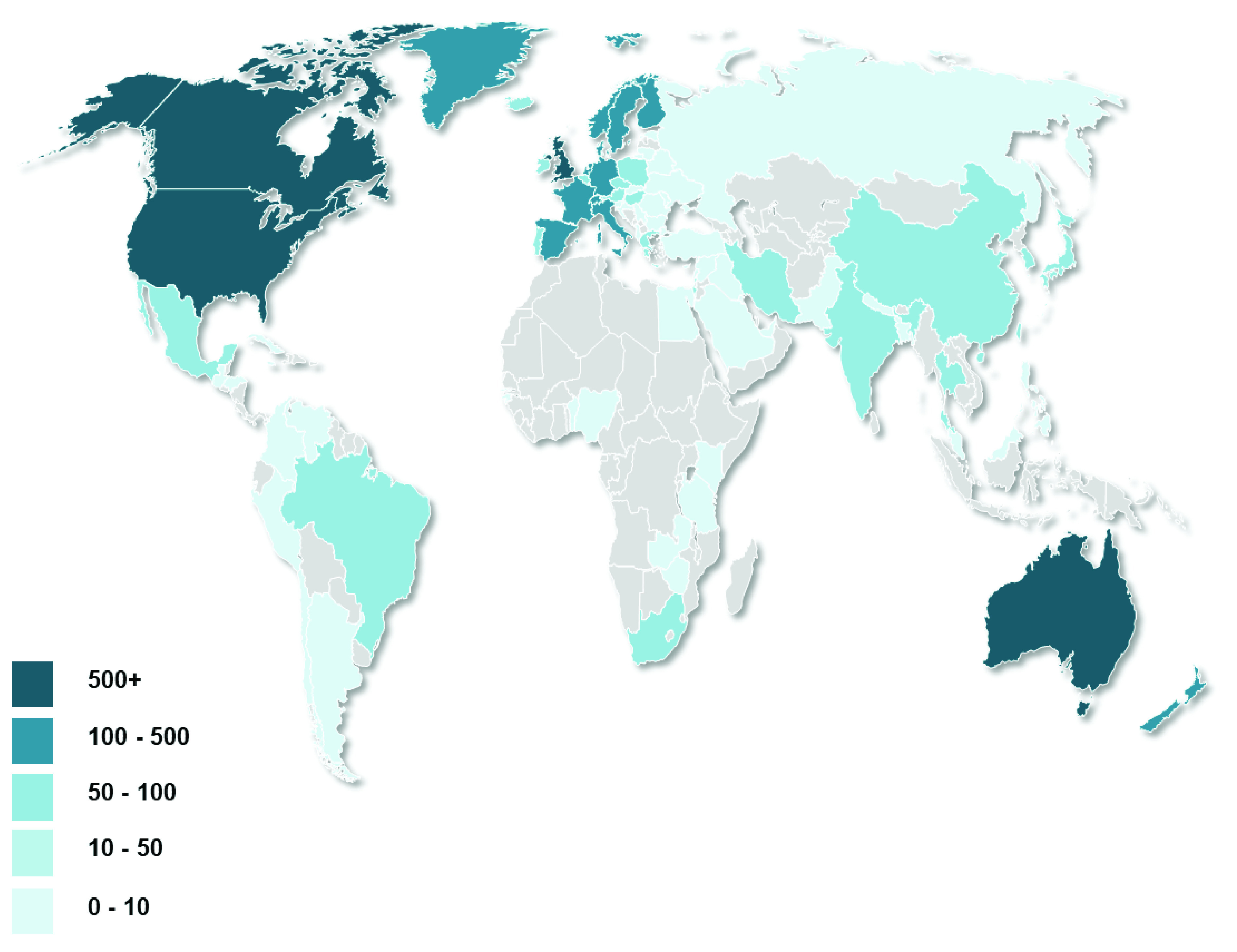
Number of papers by country institutional affiliation associated with publications in NICE public health guidelines.

We can also look at the contribution from other countries. Across the 9,391 papers, we identified 5,617 institutions across 93 countries. Figure 1 shows the distribution of institutions affiliated with the papers.

However, though many countries make some contribution, the majority of the research comes from a more limited set of countries. As shown in Figure 1, there are four countries (UK, US, Canada and Australia) who have contributed to over 500 papers, and collectively, these four countries have contributed to 90% of the papers. The largest contributor is the US - 47% of papers have a US institution affiliation (4,419). This is likely to reflect the scale of public health research conducted in the US, relative to other countries ^7, 8^.

**Figure 2.**
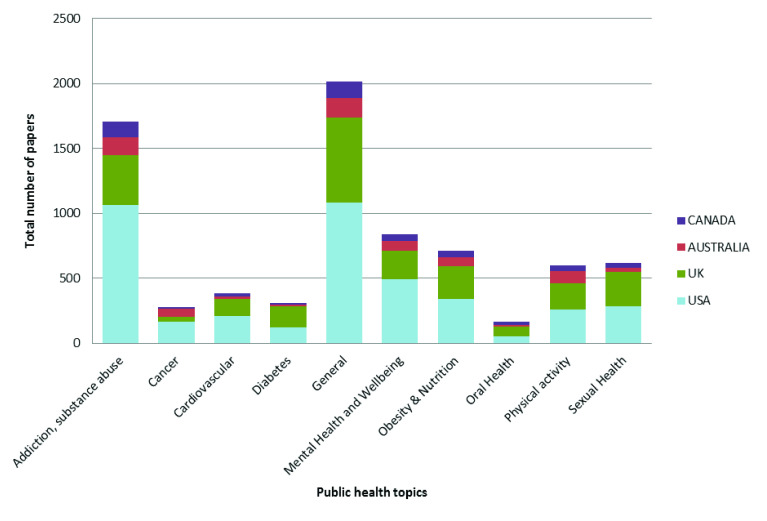
Number of papers by NICE public health guideline subject area, across four Anglophone countries.

Dividing the papers by subject area, we see that the contribution of UK research to the evidence used in guidelines is broadly similar for each subject area. Figure 2 shows the total number of papers for UK, US, Canada and Australia by public health subject area. The US accounts for the majority of papers in each subject area, with the exception of diabetes and oral health, where the UK accounts for the majority of papers (40% and 29% respectively). Papers from non-Anglophone countries account for less than 4% of papers in each subject area with the exception of: oral health, where 9% of papers are from Sweden; mental health, where 6% of papers are from the Netherlands; cardiovascular, where 4% of papers are from Finland.

**Figure 3.**
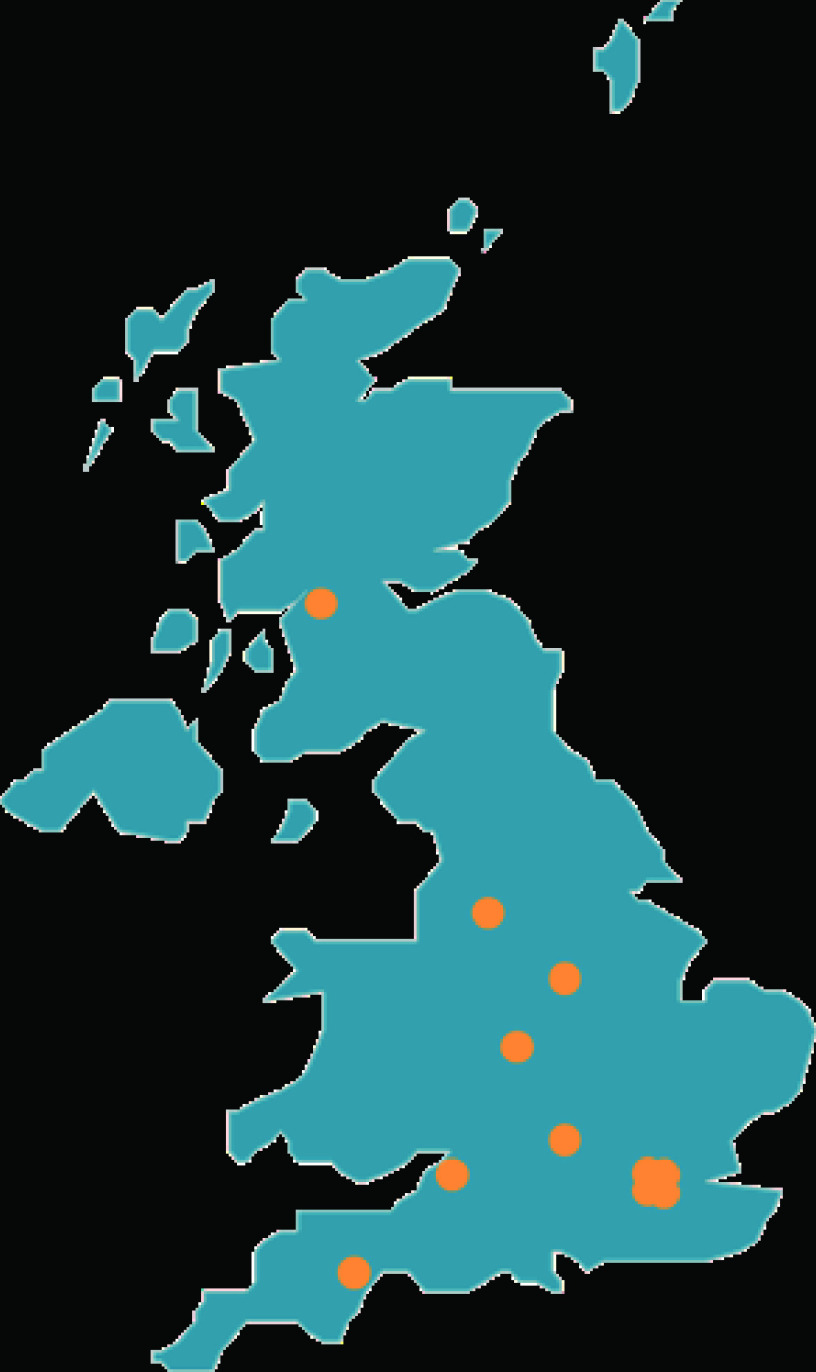
Map of top 10 UK institutions in the dataset.

We can also look at the contribution of specific institutions, which provides some scope to identify ‘centres of excellence’ in public health research. In the UK, 1,181 institutions were identified, featuring on 2,627 papers. Table 4 shows the top ten institutions from the UK in the dataset, also mapped in Figure 3.

**Table 4.** Total number of papers by UK institutional affiliation.

Academic institution	Number of papers
University College London	244
University of Bristol	176
London School of Hygiene and Tropical Medicine	158
University of Glasgow	120
University of Oxford	118
University of Nottingham	112
Kings College London	112
University of London	100
University of Birmingham	95
University of Manchester	92

**Figure 4.**
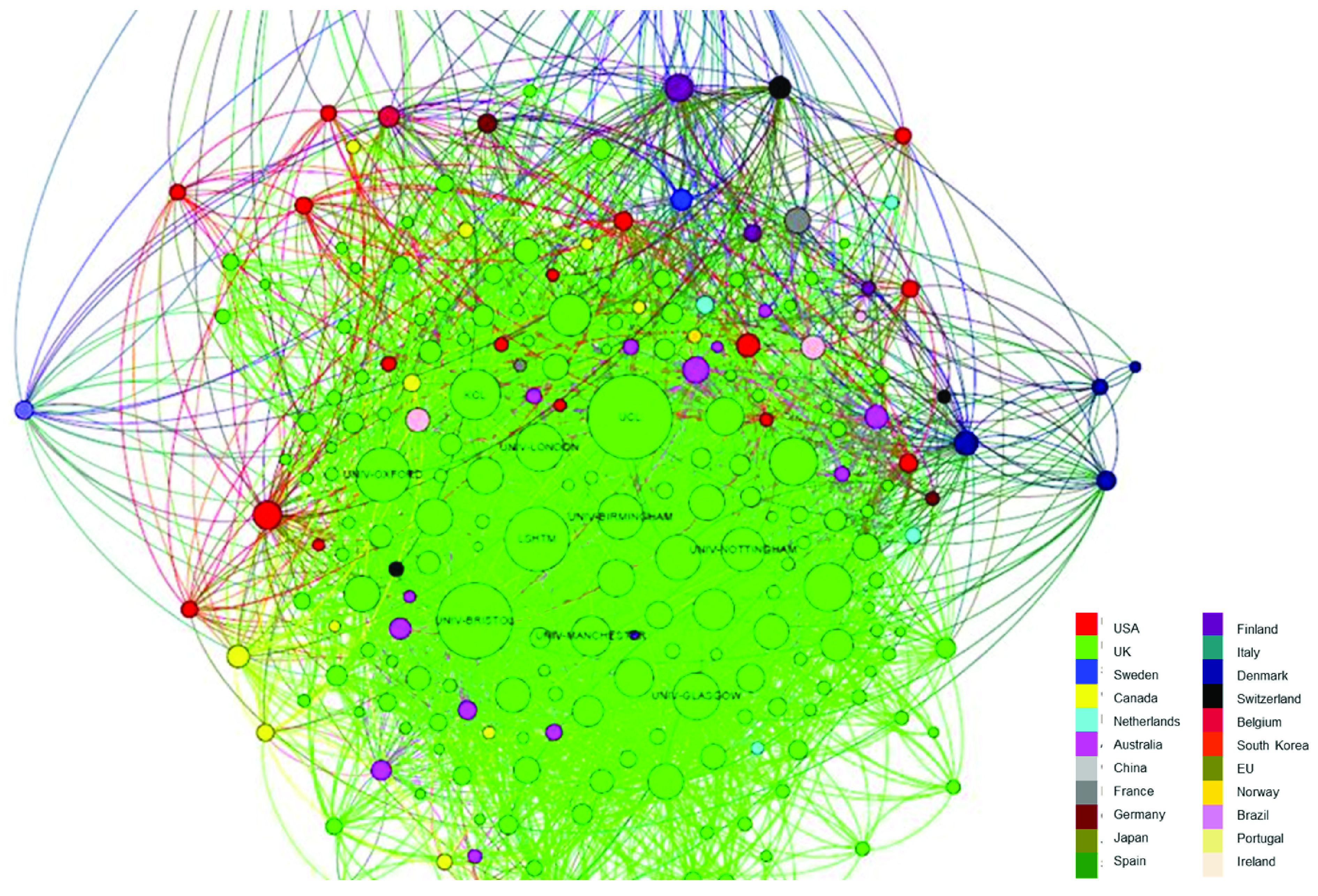
Co-acknowledgement network – institutions affiliated with papers with a UK address (n=2,627). Each node represents an institution and the connecting lines indicate these institutions’ co-acknowledgement on papers (a thicker line indicates a greater number of co-acknowledgements). The size of a node is proportional to the number of papers with a UK corresponding address affiliated to the institution. Institutions with fewer than 4 papers with an associated UK address are not included in the network. Nodes are coloured by the location of the institutions.

Figure 4 outlines the network of institutional affiliations on papers with a UK address. In the network, each node represents an institution and the connecting lines indicate these institutions’ co-acknowledgement on papers (a thicker line indicates a greater number of co-acknowledgements). The size of a node is proportional to the number of papers with a UK corresponding address affiliated to the institution. Institutions with fewer than 4 papers with an associated UK address are not included in the network. Nodes are coloured by the location of the institutions.

Aside from the top UK institutions in the network (see Table 4), institutions from other Anglophone countries, such as Australia, US, New Zealand and Canada, tend to be the most prominent non-UK institutions in the network.

Overall, the UK is an important contributor of research to the evidence base supporting NICE public health guidelines, contributing to 28% of papers, alongside other Anglophone countries. This may partly reflect biases in the WoS towards English language publications but may also reflect the strength of these countries in the field. Clarke *et al.*’s bibliometric overview of public health research in Europe ^7^ found that only 3.5% of research papers were published in non-English languages. This does not differ significantly by field of research within public health.

### Contribution of the UK as a funder of public health research

The second level of analysis was to assess the distribution and co-occurrence of recorded funding body acknowledgements (FBAs) from publications cited in the guidelines. This bottom-up approach has been used in various studies to map the funding landscape of a particular field, explore interactions among research funders and assess trends in research funding ^9– 12^.

1,420 papers contain FBAs with an average of 2.98 acknowledgements per paper. This represents 15% of the dataset, which is lower than other studies ^12^ but may reflect the fact that 67% of the papers in the dataset were published before 2008 – the year WoS began collecting data on FBAs.

Across these papers, 165 unique funders were identified with more than three acknowledgements, based in 20 countries. Table 5 shows the total number of papers and FBAs by funder location.

**Table 5.** Number of papers with funding body acknowledgements (FBAs), by country.

Location of the funder	Number of papers with FBAs	Total number of FBAs
UK	966	1073
USA	794	1056
Australia	221	244
Canada	128	148
Finland	73	89
Denmark	56	57
Switzerland	50	54
EU	50	55
New Zealand	41	45
Germany	39	38
Netherlands	37	35
Sweden	35	30
France	35	31
Japan	17	24
Spain	12	25
Norway	10	6
Belgium	8	8
China	6	4
Brazil	4	4
Portugal	3	5

While there are significantly more US-based institutions affiliated with papers in the dataset, there are more papers with UK-based funders acknowledged than the US. NIHR is the most acknowledged funder in the dataset, accounting for 11% of papers with FBAs. Taken together with the UK Department of Health, it accounts for 226 papers (16% of papers with FBAs). Table 5 shows the top ten most acknowledged funders in the dataset, which account for 60% of the papers with FBAs. 

Along with the UK Department of Health, research councils (such as MRC and ESRC) and disease-specific charities working in public health (such as British Heart Foundation and Cancer Research UK) are the most prominent UK funders in the dataset. In addition to UK funders, we see two US funders and one Australian funder in the top ten.

**Figure 5.**
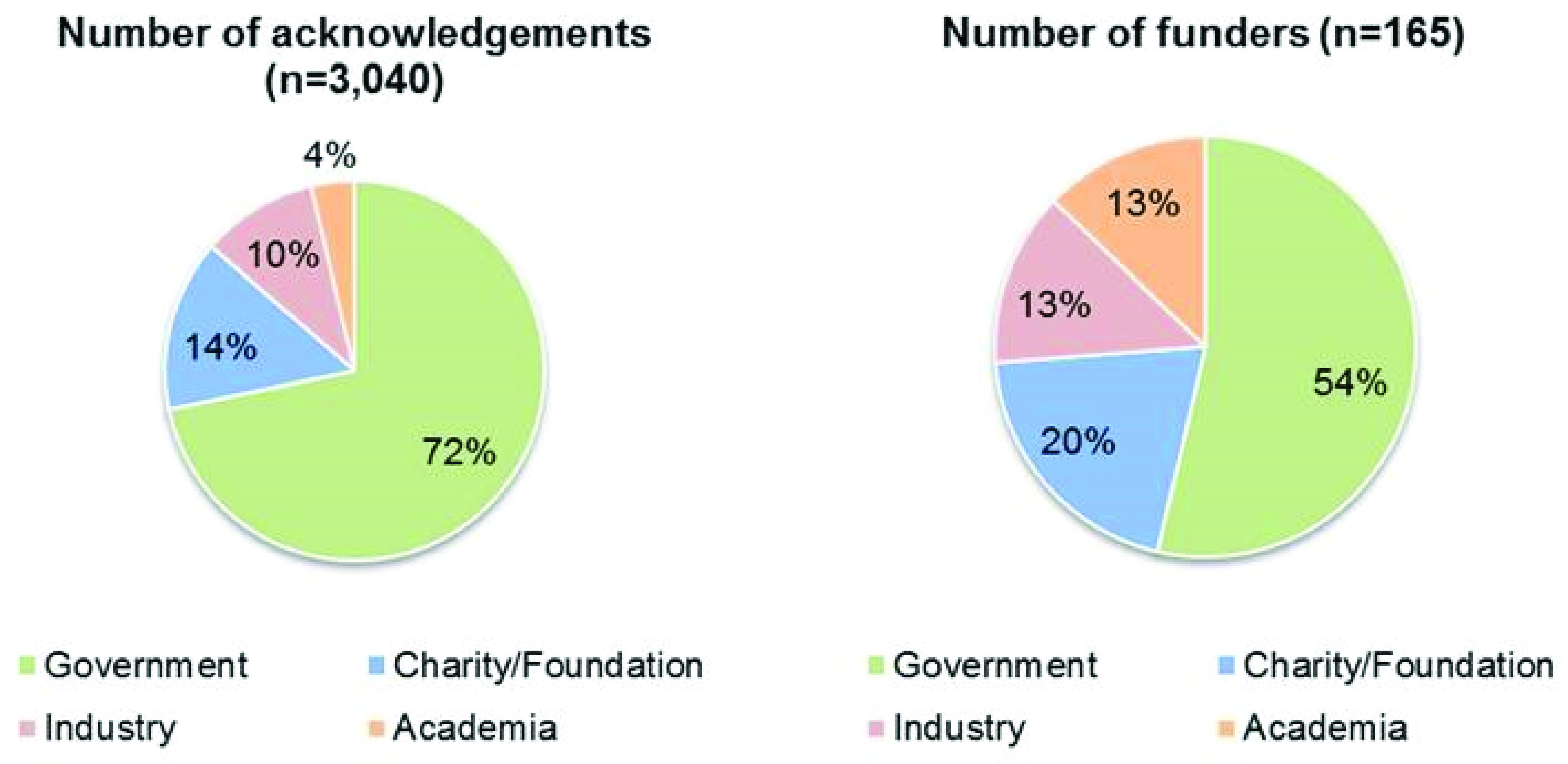
Proportion of funding body acknowledgements and funders by sector (n=1,420).

Government funders make up the majority of the 165 funders in our data set, accounting for eight of the top ten funders and around 72% of all funding acknowledgements ( Figure 5). While our previous study on the FBAs associated with global mental health research ^12^ found a similar distribution in the overall number of acknowledgements (with government funding accounting for 68%), the study found a significantly larger proportion of charities, foundations and non-profits in relation to the total number of funders.

**Figure 6.**
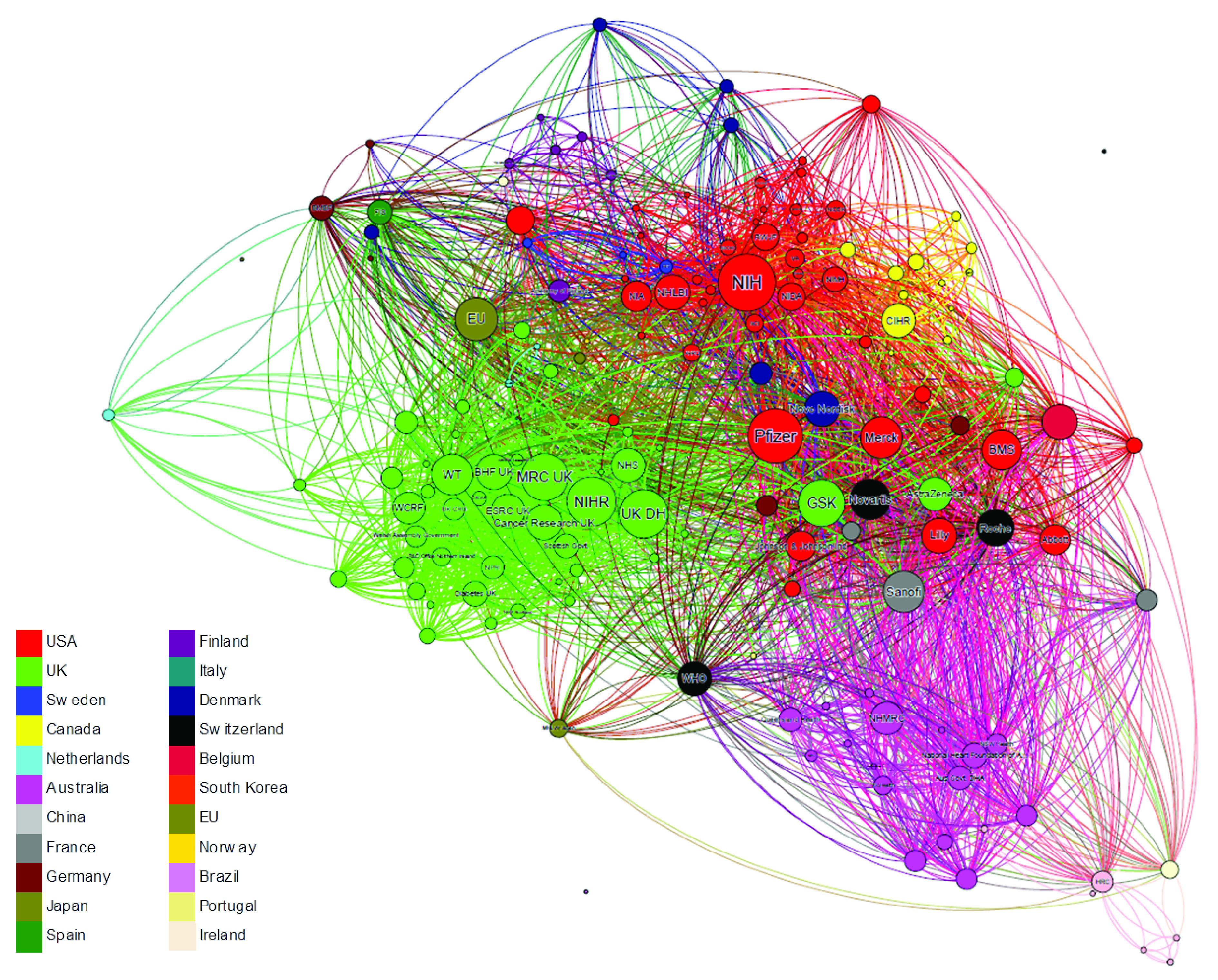
Co-acknowledgement network of funders acknowledged on all papers cited in NICE guidelines (n=1,420). Each node represents a funder and the connecting lines indicate these funders’ co-acknowledgement on papers (a thicker line indicates a greater number of co-acknowledgements). The size of a node is proportional to the number of papers in the network. Funders with fewer than 10 ten papers are not included in the network. Nodes are coloured by the location of the funder.

Figure 6 outlines the network of funders acknowledged in papers across the dataset. In the network, each node represents a funder and the connecting lines indicate these funders’ co-acknowledgement on papers (a thicker line indicates a greater number of co-acknowledgements). The size of a node is proportional to the number of papers in the network. Funders with fewer than ten papers are not included in the network. Nodes are coloured by the location of the funder.

The most prominent funders in the network are the most frequently-acknowledged US and UK funders (see Table 5). There is also a relatively large contingent from Australia, multilateral funders such as the EU and the WHO, as well as a number of pharmaceutical companies, such as Pfizer, Merck, GSK and Sanofi. Government funders in Canada, the Netherlands and Scandinavia are also visible.

Of the 165 unique funders identified with more than three acknowledgements, 43 funders (26%) are based in the UK. We have split UK government funding, which make up 42% of the UK funders, into five funder groups for the purposes of analysis.

- The “UK Department of Health”, which includes National Institute for Health Research (NIHR) and NHS England as well as the UK Clinical Research Collaboration (UK CRC) and National Institute for Health and Clinical Excellence (NICE);
- Research Councils, primarily the UK Medical Research Council (MRC), make up the majority of funding acknowledgements in the dataset. Additional research councils in the data set includes: Economic and Social Research Council (ESRC), Biotechnology and Biological Sciences Research Council (BBSRC), Engineering and Physical Sciences Research Council (EPSRC), and Arts and Humanities Research Council (AHRC);
- Devolved government departments, in Scotland, Wales and Northern Ireland;
- Non-health focussed central government departments, such as the Department for International Development (DFID);
- Non-departmental public bodies, both with a health focus (e.g. British Health and Safety Executive) and non-health focus (e.g. UK Food Standards Agency, Higher Education Funding Council for England (HEFCE)).

**Figure 7.**
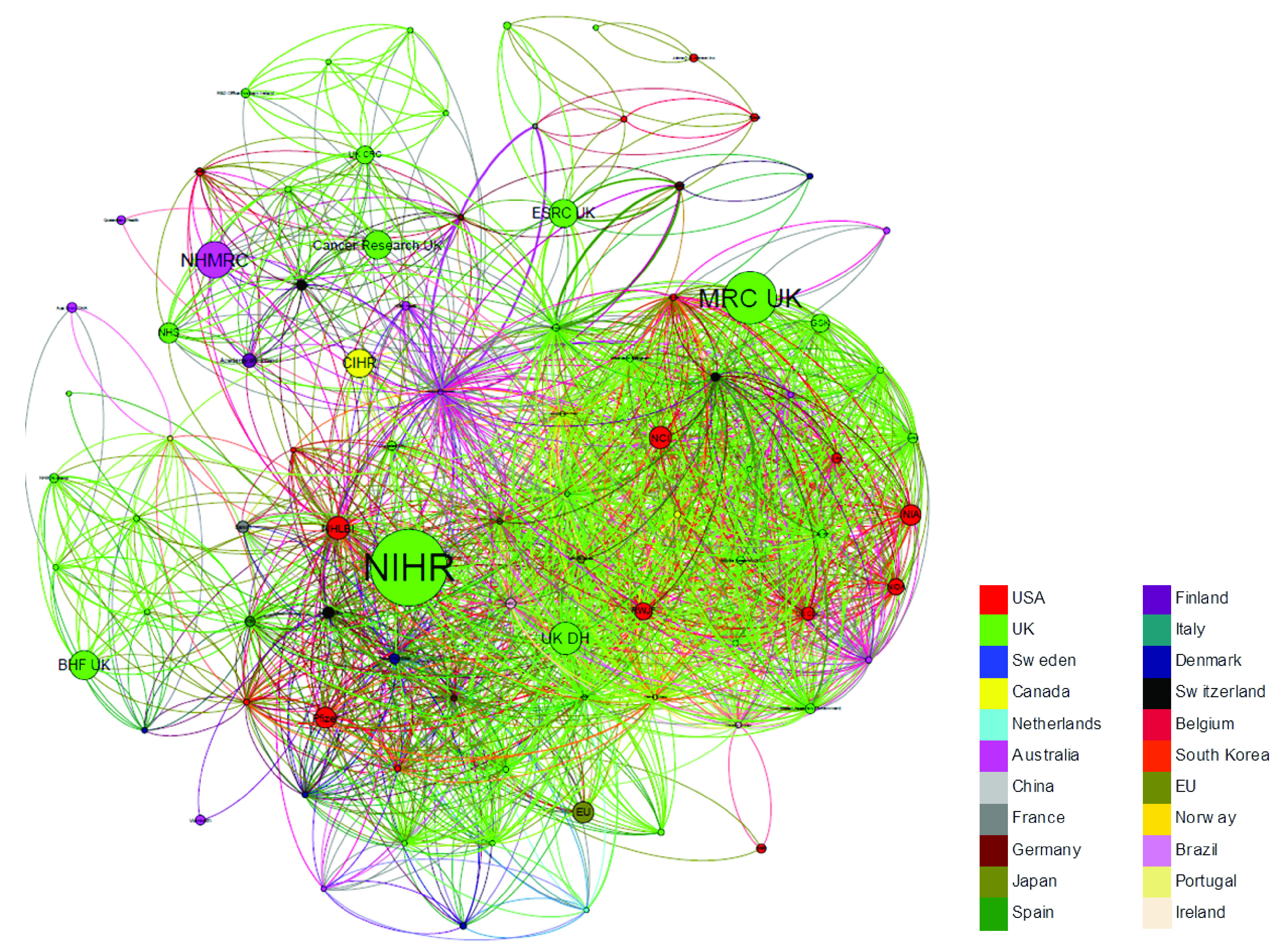
Co-acknowledgement network of funders acknowledged on UK papers cited in NICE guidelines (n=1,420). Each node represents a funder and the connecting lines indicate these funders’ co-acknowledgement on papers (a thicker line indicates a greater number of co-acknowledgements). The size of a node is proportional to the number of papers in the network. Funders with fewer than 3 papers with an associated UK address are not included in the network. Nodes are coloured by the location of the funder.

Figure 7 outlines the network of funders acknowledged on papers with an associated UK address. In the network, each node represents a funder and the connecting lines indicate these funders’ co-acknowledgement on papers (a thicker line indicates a greater number of co-acknowledgements). The size of a node is proportional to the number of papers in the network. Funders with fewer than 3 papers with an associated UK address are not included in the network. Nodes are coloured by the location of the funder.

**Figure 8.**
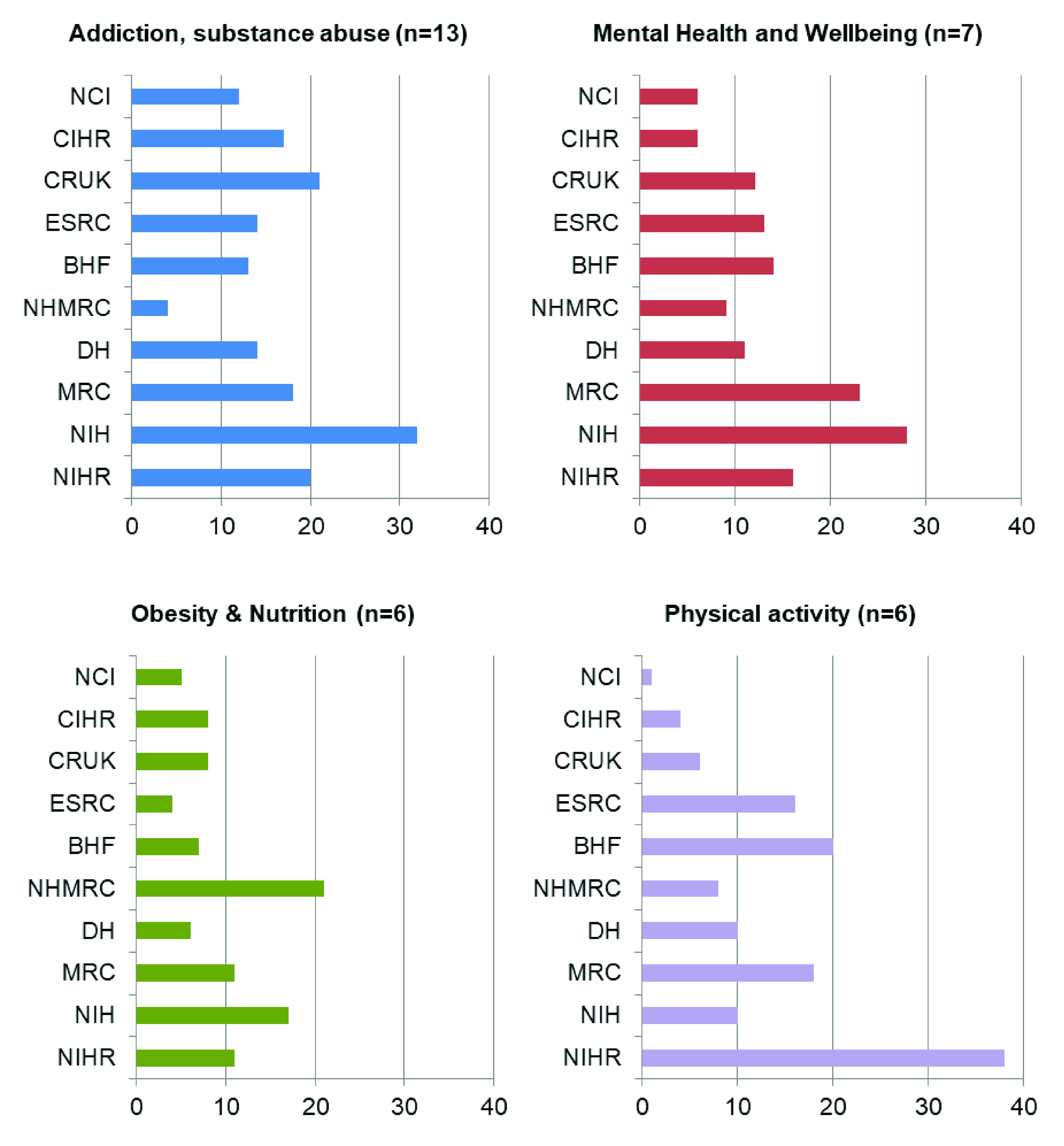
Breakdown of papers by NICE public health guideline subject area funded by top ten funders. NCI, National Cancer Institute (US); CIHR, Canadian Institutes of Health Research; CRUK, Cancer Research UK; ESRC, Economic and Social Research Council (UK); BHF, British Heart Foundation; NHMRC, National Health and Medical Research Council (Australia); DH, Department of Health (England); MRC, Medical Research Council (UK); NIH, National Institutes of Health (US); NIHR – National Institute for Health Research (England).

Breaking this down by subject area, we see that the contribution of research funders varies according to the subject area of the guideline. Figure 8 highlights the number of papers cited on the four main subject areas of NICE public health guidelines across the top ten research funders identified. For guidelines on addiction and substance abuse (of which the majority are primarily focussed on smoking), the US National Institutes of Health is acknowledged the most, followed by Cancer Research UK and NIHR. The US National Institutes of Health is also the most frequently acknowledged funder on mental health and wellbeing guidelines. However, for guidelines focussed on obesity and nutrition, the Australian National Health and Medical Research Council (NHMRC) is the most frequently acknowledged funder and for guidelines on physical activity, NIHR is the most acknowledged funder, with the top four funders all based in the UK. However, it is important to reiterate in interpreting this data that only 15% of papers in the dataset contain FBAs.

## Discussion

Based on the analysis above, we can conclude that UK public health research and research funders make an important contribution to NICE guidelines. Of the publications matched in Web of Science, 28% have at least one UK author, which is relatively high compared to other fields where such analysis has been conducted, as shown in Table 6, suggesting that context is important to the application of research to policy in this field. In addition, 68% of papers which acknowledge funding cite at least one UK funder, and NIHR is the most highly cited funder in the sample. This suggests that UK funded research in particular is important here, perhaps since research priorities are likely to correspond to policy and practice needs in the UK context. However, there may be other reasons for this – for example, it may be that UK funders are more likely to require acknowledgement of their funding on publications, or that the more recent papers, which are more likely to have funding acknowledgements, are more likely to be from UK sources. This may make sense, as this may correspond to more recent research that reflects the implementation of research in practice, building on a wider body of research which could come from a wider range of sources and countries, since the contextual factors are less crucial to the relevance of this broader underpinning body of knowledge.

**Table 6.** Contribution of UK research to specific guidelines.

Field	Attribution to UK	Source
Cancer	17%	Estimating the returns to UK publicly funded cancer-related research in terms of the net value of improved health outcomes Matthew Glover, Martin Buxton, Susan Guthrie, Stephen Hanney, Alexandra Pollitt and Jonathan Grant BMC Medicine 2014 12:99
Cardiovascular	17%	Health Economics Research Group, Office of Health Economics, RAND Europe. Medical Research: What’s it worth? Estimating the economic benefits from medical research in the UK. London: UK Evaluation Forum; 2008.
Mental health	28%	Health Economics Research Group, Office of Health Economics, RAND Europe. Medical Research: What’s it worth? Estimating the economic benefits from medical research in the UK. London: UK Evaluation Forum; 2008.
Musculoskeletal	30%	Estimating the returns to UK publicly funded musculoskeletal disease research in terms of net value of improved health outcomes Matthew Glover, Erin Montague, Alexandra Pollitt, Susan Guthrie, Stephen Hanney, Martin Buxton and Jonathan Grant BMC Medicine (submitted)

Although we can make the case that UK public health research does make an important contribution to NICE guidelines, the extent to which this reflects practice, and hence the extent to which UK public health research can be said to be influencing practice in the UK, is less clear. Based on the interviews we conducted, the extent to which NICE guidelines are seen to reflect practice varies significantly, depending on the prevailing circumstances of each local authority area. NICE guidelines on some aspects, such as obesity, smoking cessation, children’s health and cancer prevention, are considered relevant across the board. However, due to budgetary reasons, statutory requirements and geographical variations, local practice can differ significantly (INT01; INT03; INT04; INT08; INT10; INT11).

Since the politics of representation and the principle of democratic accountability are crucial to the functioning of local authorities, NICE guidelines, although considered, are not always the definitive evidence for commissioning or recommissioning services (INT02; INT08; INT10). The extent to which the guidelines are deemed to reflect practice also depends on the commissioning / recommissioning cycles. Multiple interviewees argued that NICE guidelines can be considered crucial in relation to evaluating existing services and whether the services meet the requirements set by the guidelines (specifically if budget cuts are to be achieved) (INT01; INT03; INT05; INT11).

Depending on how the local authorities understand and interpret the role of NICE guidelines, the perceptions of the extent to which NICE guidelines reflect practice varies. Multiple interviewees highlighted the need to communicate more clearly that NICE guidelines are only meant to help make a public health-related decision and not present a ready-made decision on the behalf of the authorities (INT02; INT03; INT04; INT05; INT08; INT10). These interviewees contended that NICE guidelines are aimed at achieving national relevance and the extent to which some of the guidance can be implemented in the context of specific local challenges would need to vary accordingly. This is reflected in how local authorities with predominantly rural and semi-urban areas with sparse populations are likely to diverge in their approach when considering the guidelines to be implemented (INT04; INT05; INT06; INT08). The following quote elaborates on how the needs of rural, semi-urban areas differ when compared to urban areas. The quote has been paraphrased to protect the anonymity of the interview participant:

*“… It’s a common problem [the urban focus] that we are discussing with Public Health England. Identifying the requirements for rural community [is a challenge]. If you look at sexual health guidelines (HIV, syphilis etc.), the outreach worker in rural areas sometimes has to travel 5 times the distance than the one in central London. How do you adapt service models to that? Some of the NICE guidance refers to smartphone apps and relies on people having access to the Internet, whereas in the rural areas, superfast broadband is non-existent. ” [INT04]*

Given the constraints of budget cuts / austerity, more than one director of public health mentioned that they sought evidence from other (non-UK) countries in relation to their decision-making for local public health priorities (INT04; INT06). Since there is insufficient precedent for the use of such evidence, the interviewees also suggested that guidance on weighing the appropriateness of the evidence for a particular local authority context would be useful. Such guidance was perceived as being potentially useful to practice and decision-making at local authority area. The way NICE guidelines are perceived to consider hierarchy of evidence also forms an important part of their use in practice (INT11). The type of evidence (based on Randomised Control Trials i.e. RCTs, modelling in some cases) varies and some interviewees suggested that as part of recommendations, the level of evidence is not always identified. This can pose challenges to local authorities when deciding their needs for evidence or additional evidence synthesis. For evidence deemed high-level and not necessarily based on detailed case studies, the NICE guidelines were often considered to be less reflective of the actual practice by some of the interviewees (INT01; INT03; INT04; INT11).

An important document at the local authority level is the health and wellbeing strategy. In recognising that NICE guidelines focus on treatment and prevention measures, local authorities emphasise engagement with local stakeholders and locally produced intelligence when creating and updating health and wellbeing strategies. Given that the local authorities are best placed to gather and act upon local intelligence, multiple interviewees suggested that the upstream for local authorities into planning and regulation related to public health decision-making needs further consideration. According to some interviewees, such upstream input could strengthen the guidelines and their use in practice (INT03; INT04; INT05). The following quote provides further insight into this position:

*“We have an input into the development of national policy. I am not sure how much it determines what national do, because their work is more centralised. You do find situations when national policy and recommendations differs from the challenges at the local level authority.*
*What national does is great if it feeds into what happening locally. But differences can exist from what the national agenda suggests. I am not sure how much we influence the national agenda. We certainly try.” [INT05]*

An alternative approach to look at the impact of research on practice in public health would be to analyse documentation at the local authority level. However, based on the interviews and desk research we conducted, there are likely to be many challenges with this alternative approach:

- The local authorities follow different processes and approaches to document and store their approach and policies for public health-related decision-making. This makes it time-consuming to track and identify local authorities’ public health-related documentation.
- Although there is a degree of consistency in terms of storing documentation related to the Joint Strategic Needs Assessment (JSNAs), the practices in terms of citations and referencing vary significantly. with many documents setting out strategy including no formal referencing of research or underpinning evidence.
- In addition to the different practices for referencing and citation of the evidence, the use of evidence at the local level (including local statistics, local survey data and local population data) varies significantly. The citation and referencing of local evidence in this form varies, which makes the analysis of local authorities’ documentation a time-consuming task.
- Document sharing with local government associations varies and the processes can often be informal in nature, which makes it difficult to authoritatively establish the reach and impact of the documentation produced by the local authorities.
- Document sharing between local authorities varies based on local areas and extent of coordination and leadership approaches are also different. This has an influence on the documentation publicly available and the manner in which evidence is cited.
- Given the emphasis on evidence-based decision-making in case of some priorities, evidence gathered and analysed by the teams that support the DPH and the documentation is internally provided. This evidence may not be formally cited depending on the nature of documentation which may / may not be available publicly.
- The public availability of the documents is shaped by contracting decisions and whether recommissioning is necessary in relation to a public health service. Consequently, making a realistic assessment of the evidence included in documentation related to public health was not feasible in the timescales allocated for this research.

### Limitations of the study

As noted above, a key limitation of this analysis is the extent to which we can link citation on NICE guidelines to changes in practice. In some areas of health research there are reasonable grounds to assume that practice should, at least to some extent, reflect, or be moving towards, best practice, as set out in guidelines. However, this is less clear in public health, where implementation can take multiple routes and is very much local-context dependent. In addition, we can also add the usual caveats around the use of bibliometrics, some of which are clearly emphasised here. Firstly, only a relatively small proportion of the publications from the guidelines were matched in Web of Science, which may be because much of what is cited on guidelines are not journal publications. This means the analysis here is only a reflection of that portion of the evidence used to underpin guidelines. Secondly, there are a number of known biases in the coverage of bibliometric databases – notably in relation to language, where English language publications are more covered than other languages, which may bias towards publications from the UK and other Anglophone countries. Finally, it should be noted that a full counting approach is used in the analysis here, meaning we look at the number of papers to which researchers or funders from a particular country has contributed. For many of these, there will also be contributions from other countries, and this analysis cannot account for the extent of the contribution of the different authors and/or funders, which may differ substantially.

In terms of the interviews conducted, we are of course limited to the range of viewpoints provided by the set of individuals to whom we spoke. There may be perspectives which we did not capture, and there may be biases inherent of the sample of individuals willing to speak about their experience and knowledge of evidence supporting practice in public health. However, we conducted sufficient interviews that we reached ‘saturation point’, that is, additional interviews did not seem to be adding substantially to the range and nature of information captured. We also compared the interview findings to wider published evidence on the topic.

## Conclusions

UK public health research makes an important contribution towards NICE guidelines, accounting for 28% of all cited papers. Research is broadly collaborative and coordinated across funders within the UK and in this respect, public health as a research field does not appear to be bibliometrically distinct from other research sectors. As the largest health research funder in the UK, it is also not surprising that the NIHR is the most acknowledged funder on papers cited in NICE public health guidelines.

There is, however, a relatively low-level of international collaboration on UK papers cited in the guidelines. Given the nature of the dataset, this may be expected for two reasons. Firstly, 23% of the papers included in the guidelines were published before the year 2000, when levels of international collaboration were lower. Secondly, the interviews conducted with public health experts highlight the local nature of public health research, which may mean that international collaboration is less likely in these fields.

How far NICE public health guidelines reflect practice conducted at the local authority level is less clear. Therefore, the extent to which public health research truly drives practice is harder to determine. More research is needed to understand the sources of evidence to support public health decision making at the local level, and how NICE guidance can be made more applicable, timely and accessible in this new context.

## Data availability

### Underlying data

 Interviews were conducted under the principle of informed consent. Interview participants were informed that recordings, transcripts and notes from the interviews were solely for the use of the project team and therefore these cannot be made publicly available, even in an anonymised form. If researchers wish to access these notes for the purposes of further research, they may contact the corresponding study author, Dr Susan Guthrie, by email ( sguthrie@rand.org), providing details of the information required and the intended use of the data. Subject to approval by the RAND Human Subjects Protection Committee, we will then contact the interview participant(s) in question to seek their permission to share the interview notes for the purpose specified.

Figshare: Papers from NICE public health guidelines matched in Web of Science.csv. https://doi.org/10.6084/m9.figshare.8053187.v1 ^13^

This project contains the following underlying data:

- Papers from NICE public health guidelines matched in Web of Science.csv (List of papers cited on NICE public health guidelines which were matched in Web of Science)

Data are available under the terms of the Creative Commons Zero "No rights reserved" data waiver (CC0 1.0 Public domain dedication).
