# Supplementary material for: Understanding the contribution of UK public health research to clinical guidelines: a bibliometric analysis
Source: F1000Res. 2019 Jul 15;8:1093. [Version 1] doi: 10.12688/f1000research.18757.1 (PMC7845157; doi:10.12688/f1000research.18757.1)
Supplement: Peer review history

# Peer review: Reviewer response for version 1

**DOI:** 10.5256/f1000research.20547.r77488

**Published:** 2021-01-28

**Authors:** Andrea Tittarelli, Claudio Tresoldi

**Affiliations:** 1 Fondazione IRCCS Istituto Nazionale Tumori, Milano, Italy; 2 Department of Research, Cancer Registry Unit, Fondazione IRCCS Istituto Nazionale dei Tumori di Milano, Milano, Italy

The paper is unusual and addresses a particular topic, maybe more difficult for a reader outside UK, but it is however interesting and well done, presenting useful and remarkable results.

 However, some minor adjustments are recommended to improve it.

**Specific points:**

**Abstract**

- The acronyms NIHR and PH here are not defined.

**Introduction**

- The acronym NHS is not defined.

**Methods - *Analysis of references cited on NICE guidelines***

- There is no reference for software R; in scientific publications the following is usually used:

*R Core Team (2020). R: A language and environment for statistical computing. R Foundation for Statistical Computing, Vienna, Austria. URL https://www.R-project.org/*

**Results - *Contribution of the UK as a funder of public health research***

- The second reference to Table 5 (Page  8, line 21 of that paragraph: “ *Table 5 shows the top ten most acknowledged funders in the dataset, which account for 60% of the papers with FBAs*”) seems not pertinent, as in Table 5 there is not a list of funders.

**Discussion**

- Page 14, penultimate line of left column: the acronym DPH is not defined.

**Tables**

- Table 2: there are acronyms used without any previous definition, i.e. LAs, CCGs, PH.

**Figures**

- Figure 1: grey color is not defined in the legend; it is supposed to indicate countries counting zero contributing papers, but this is contradictory with the group represented by the lightest blue, which seems to include also the 0.
- Figure 4 is really too complex and hard to understand! Thicker lines are difficult to distinguish. Moreover, it is also cut at the top and the bottom.
- Figure 6 and 7 are less confuse than Figure 4, but thicker lines are still hard to distinguish.
- Figure 8: it is not clear the meaning of the numbers **n** in brackets after each of the four headings.

Is the work clearly and accurately presented and does it cite the current literature?

Yes

If applicable, is the statistical analysis and its interpretation appropriate?

Yes

Are all the source data underlying the results available to ensure full reproducibility?

Yes

Is the study design appropriate and is the work technically sound?

Yes

Are the conclusions drawn adequately supported by the results?

Yes

Are sufficient details of methods and analysis provided to allow replication by others?

Yes

Reviewer Expertise:

Epidemiology, Statistics

We confirm that we have read this submission and believe that we have an appropriate level of expertise to confirm that it is of an acceptable scientific standard.

# Peer review: Reviewer response for version 1

**DOI:** 10.5256/f1000research.20547.r51248

**Published:** 2019-07-17

**Authors:** Mike Thelwall

**Affiliations:** 1 Statistical Cybermetrics Research Group, University of Wolverhampton, Wolverhampton, UK

This is a descriptive analysis of references in UK NICE guidelines using bibliometrics together with stakeholder interviews for context. Although clinical guideline citations have been investigated before, I think this is the first to match citations with the web of science and to analyse them in conjunction with interviews. The paper contains a lot of results, is well written and clearly explained. The interviews do not seem to have been systematically analysed with a recognised qualitative method but I think this is fine given that they are used to support and follow up from the bibliometric analysis, as well as to address pre-defined questions. It also does not have a literature review component summarising what is known about NICE guidelines from previous research but it does not really need it.

 For the sentence, “This is below the general average for international collaboration in research across the UK”, could you find from the Web of Science what the average is for public health? If not, the science-wide comparison is not very meaningful.

Is the work clearly and accurately presented and does it cite the current literature?

Yes

If applicable, is the statistical analysis and its interpretation appropriate?

Yes

Are all the source data underlying the results available to ensure full reproducibility?

Partly

Is the study design appropriate and is the work technically sound?

Yes

Are the conclusions drawn adequately supported by the results?

Yes

Are sufficient details of methods and analysis provided to allow replication by others?

Yes

Reviewer Expertise:

Bibliometrics

I confirm that I have read this submission and believe that I have an appropriate level of expertise to confirm that it is of an acceptable scientific standard.
